# The Justification of Open Surgical Repair for an Abdominal Aortic Aneurysm: A Retrospective Comparison of Outcomes of Endovascular Aneurysm Repair and a Brief Review of the Literature

**DOI:** 10.3390/life15030426

**Published:** 2025-03-08

**Authors:** Ümit Arslan, Ziya Yıldız, İbrahim Pir, Çağrı Aykut

**Affiliations:** 1 Department of Cardiovascular Surgery, Faculty of Medicine, Atatürk University, Erzurum 25030, Turkey; ibrahimpir29@gmail.com (İ.P.); cagriaykut17@gmail.com (Ç.A.); 2 Department of Cardiovascular Surgery, Erzurum City Hospital, Erzurum 25040, Turkey; ziyayildiz1976@gmail.com

**Keywords:** abdominal aortic aneurysm, EVAR, open surgery repair, conversion to open surgery, rupture, reinterventions

## Abstract

**Background:** Abdominal aortic aneurysms (AAAs) are life-threatening conditions that require timely intervention to prevent rupture. Endovascular aneurysm repair (EVAR) is preferred due to faster recovery and lower perioperative risk; however, intraoperative failure and long-term complications highlight the continued significance of open surgical repair (OSR) and the need for improved risk assessment. **Methods:** This retrospective study analyzed data from 210 patients who underwent EVAR (*n* = 163) or OSR (*n* = 47) at a single center. Clinical characteristics, complications, reintervention rates, and 30-day mortality were recorded. EVAR-to-OSR conversion and mortality predictors in AAA treatments were identified. **Results**: The overall mortality rate was 9.5% (20/210 patients), with 12 patients (7.3%) in the EVAR group and 8 patients (17%) in the OSR group (*p* = 0.085). Five patients required early and six required late conversion to open surgery. In follow-ups beyond 30 days, the reintervention rate for EVAR was higher (HR: 1.2, 95% CI: 0.4–3.6; *p* = 0.754). According to the multivariable analysis, rupture (*p* = 0.045), female sex (*p* = 0.018), body weight (*p* = 0.003), and aortic size index (*p* = 0.019) were significant predictors of mortality, whereas OSR was not (*p* = 0.212). **Conclusions**: Treatment optimization requires a balanced approach, integrating both EVAR and OSR based on patient-specific factors. Maintaining expertise in both techniques is essential to ensure the best possible outcomes, and OSR should remain a viable option when clinically indicated.

## 1. Introduction

Abdominal aortic aneurysms (AAAs) typically progress insidiously and remain asymptomatic until rupture, posing a substantial risk of fatal outcomes. A thorough understanding of the associated risk factors and a precise, evidence-based management strategy are imperative to mitigate these risks [1]. Despite significant advancements in tissue engineering, current AAA treatments remain confined to open surgical repair (OSR) and endovascular aneurysm repair (EVAR). Tissue engineering aims to develop biomaterials that mimic native tissue function, encompassing growth, self-repair mechanisms, and structural integrity. As a cornerstone of regenerative medicine, it integrates biological sciences, material engineering, and biomedical technologies to synthesize in vitro proteins or biomimetic cells. These cutting-edge approaches directly target pathological mechanisms within the aortic wall, with the potential therapeutic effects including the prevention of smooth muscle cell apoptosis, extracellular matrix remodeling, and the regeneration of structural aortic fibers. In this regard, nanotechnology-driven scaffolds and biomaterials hold considerable promise for enhancing cellular interactions, improving graft integration, and optimizing vascular tissue regeneration [2]. Over the past three decades, EVAR has gained prominence as the preferred treatment modality, initially demonstrating promising short-term outcomes. However, accumulating evidence suggests a decline in its long-term efficacy [3]. Given the substantial need for secondary interventions—including both endovascular and open surgical procedures—to address AAAs-related complications, it is essential that treatment centers are equipped with state-of-the-art facilities and staffed with highly specialized professionals [4]. Complications, including persistent aneurysm sac expansion, endoleaks, graft infections, graft migration, and thrombosis, occur in approximately 5–10% of EVAR patients, often necessitating secondary open surgical interventions—procedures associated with a significantly increased mortality risk [5,6]. In the era of EVAR, maintaining proficiency in open surgical techniques remains essential for managing complex and unpredictable scenarios [7]. These challenges encompass young patients requiring durable long-term solutions, constraints related to material availability, and complications arising from stent graft failure or complex aneurysm anatomies. This study aimed to compare the clinical outcomes of EVAR and OSR, including conversions from EVAR to OSR, while integrating supporting visual materials and a concise review of the relevant literature.

## 2. Materials and Methods

### 2.1. Study Design

This study was conducted as a retrospective cohort analysis and was supplemented by a brief literature review to provide contextual background and strengthen the discussion. To identify relevant studies, systematic searches were performed in PubMed, Scopus, and Web of Science (WoS) using the following keywords: “abdominal aortic aneurysm (AAA)”, “endovascular aneurysm repair (EVAR)”, “open surgical repair (OSR)”, “conversion to open surgery (COS)”, and “reintervention”.

### 2.2. Participants and Data Collection

Between 2018 and 2023, data from 210 consecutive patients diagnosed with AAA and treated with EVAR (*n* = 163) or open surgery (*n* = 47) at a single center were retrospectively analyzed. Patient information was extracted from a hospital-based medical and administrative database, which is prospectively maintained by physicians and systematically records clinical data. This database includes comprehensive records on patients’ clinical characteristics, performed surgeries, radiological imaging, consultation notes, and treatments administered throughout their intensive care unit and ward stays. Additionally, all anamnesis and other clinical details are archived in physical records. For patients who were contacted and had undergone follow-up examinations or treatments in other hospitals, whether related to aneurysm management or not, all relevant medical records were retrieved via the Turkish Ministry of Health’s e-Nabız (https://enabiz.gov.tr) health portal with the patients’ consent. To minimize missing data, patients who could not be reached by phone or declined follow-up were excluded from the study. The dataset was reviewed and found to be complete, with no substantial missing data affecting the analysis. The collected data included the rupture status and comorbidities such as hypertension, diabetes mellitus, chronic obstructive pulmonary disease (COPD), coronary artery disease, kidney disease, cancer treatment, autoimmune disorders, and liver dysfunction. (In the acute phase, liver dysfunction was defined as a ≥3-fold increase in AST and ALT levels, whereas in the chronic phase, it was characterized by persistent enzyme elevation for >2 weeks or ongoing hepatology follow-up [8,9]). Smoking history, prior cardiovascular surgeries, and hospitalizations were documented to ensure a comprehensive clinical assessment.

Emergency or elective computed tomography (CT) scans were reviewed to measure the anterior–posterior and transverse diameters of the AAA at its widest point, as well as the diameters at the level of the renal arteries. These measurements were manually performed by cardiovascular surgeons using both a 3D Slicer (Harvard University, Cambridge, MA, USA) with centerline reconstruction and a RadiAnt DICOM Viewer (Medixant, Poznań, Poland), following standardized imaging protocols to ensure accuracy and consistency. At the level of the renal arteries, the aorta represents the proximal landing zone for stent graft fixation during EVAR procedures. This diameter is particularly important in our center due to the use of grafts with suprarenal fixation. The body mass index (BMI) was calculated as the weight (kg) divided by the height squared (m^2^). The body surface area (BSA) was determined using the Du Bois and Du Bois formula: [BSA = 0.007184 × height (m) 0.725 × weight (kg) 0.425] The aortic size index (ASI) was calculated by dividing the transverse aortic diameter (cm) by the BSA (m^2^) [10].

### 2.3. Interventions and Outcomes

The procedure type (EVAR or OSR) and surgery duration—defined as the time from the initial incision to wound closure—were recorded. EVAR-related interventions, including ballooning, graft extensions, and extra-anatomic bypasses, were documented. Complications such as bleeding, reoperation (e.g., for abdominal tamponade or drainage), wound infections, hypotensive shock, lower-extremity ischemia, graft thrombosis, myocardial infarction, cerebrovascular events, pseudoaneurysm, acute renal failure, and contrast-induced nephropathy were systematically recorded. Acute renal failure was defined as a ≥1.5-fold increase in serum creatinine and/or a urine output <0.5 mL/kg/h or anuria [11], while contrast-induced nephropathy was characterized by a ≥25% increase in serum creatinine within 48–72 h or the need for hemodialysis. Deaths within 30 days postoperatively were classified as 30-day mortality. Reinterventions were classified as early (<30 days) or late (>30 days) and included procedures for endoleaks, graft infections, thrombosis, stent fracture, stent graft separation, pseudoaneurysm, distal embolization, and graft migration in EVAR patients, as well as reoperations in OSR patients. Endoleaks were diagnosed via CT as contrast opacification within the aneurysm sac, but outside the stent. Stent graft separation or disjunction, particularly involving iliac limb grafts, was identified as a loss of modular integrity on CT imaging. Stent migration was defined as a ≥10 mm displacement from the original implantation site [12]. Graft infections were diagnosed based on the presence of air around the stent graft or within the aneurysm sac, accompanied by clinical symptoms.

### 2.4. Follow-Up

The follow-up duration for each patient was determined based on their last visit to the cardiovascular surgery outpatient clinic. For those who did not attend follow-up visits, attempts were made to contact them to assess their status and encourage reevaluation. If return was not possible, information was obtained to confirm whether they were alive, receiving care at another facility, or deceased. CT images from outpatient visits, along with Doppler ultrasound results when available, were reviewed. Additionally, patient records were examined for the ongoing treatment of cancer, coronary artery disease, or kidney disease. In cases of mortality, the date and cause of death were recorded.

### 2.5. Exclusion Criteria

Patients who underwent a monoiliac stent graft implantation, requiring both EVAR and open surgical intervention, as well as those treated with multilayer stent grafts for thoracoabdominal aortic aneurysms, were excluded. Additionally, patients lost to follow-up, those who were unreachable, or individuals who declined treatment or died before the intervention were not included. Patients who underwent fenestrated EVAR were also excluded.

### 2.6. EVAR Technique

All procedures were performed in a hybrid operating room equipped with a C-arm fluoroscopy system. The choice of anesthesia was based on a preoperative CT assessment of aneurysm morphology; local anesthesia with sedation was used for standard cases, while general anesthesia was preferred for complex cases. Following bilateral femoral artery exploration, intravenous heparin was administered to achieve an activated clotting time (ACT) >200 s. With the operating table and patient kept stationary, angiographic renal artery marking was performed, and the stent graft main body was deployed at a systolic blood pressure of 80–90 mmHg. Iliac extension grafts were placed whenever possible while preserving the internal iliac arteries, based on the CT evaluation. Completion angiography was conducted to assess the stent positioning, confirm adequate aneurysm exclusion, and detect potential complications such as endoleaks, stent migration, or kinking. Coil embolization, vascular plug placement, and balloon angioplasty were performed when necessary. The mean contrast volume per patient ranged from 30 to 50 mL, and the fluoroscopy time varied between 30 and 60 min (the mean value obtained from fluoroscopy was 10 mGy/minute). A single type of stent graft was used, as only suprarenal fixation PTFE (polytetrafluoroethylene) stent grafts were available. Intravascular ultrasound (IVUS) was not utilized due to unavailability at our institution.

### 2.7. Open Surgical Repair Technique

All patients underwent surgery under general anesthesia, via either a median laparotomy (preferred in rupture cases) or a retroperitoneal approach for complex AAAs. Autotransfusion, an aortic occlusion balloon, and a Foley catheter were prepared preoperatively. Central venous catheterization was performed in all patients, while a temporary hemodialysis catheter was used in rupture cases. Additionally, radial or brachial arterial catheters and a nasogastric tube were inserted. The aortic cross-clamping (ACC) site was preoperatively determined based on CT imaging, and silk tapes were placed around the proximal aorta and iliac arteries for enhanced control. Infrarenal ACC was generally preferred; however, in cases requiring suprarenal ACC, renal perfusion was maintained via a heparinized line from the brachial artery to the renal artery or a renal perfusion cannula, preventing the need for renal autotransplantation [13].

A Dacron-woven bifurcated graft was used for aortic reconstruction, with the proximal anastomosis reinforced using bovine pericardium or teflon felt. The aortobiiliac technique was the primary approach; however, in cases of iliac stenosis, occlusion, or aneurysmal involvement, an aortobifemoral bypass was performed. When the internal iliac artery was patent, it was anastomosed to the graft in an end-to-side configuration, and the inferior mesenteric artery was reimplanted to maintain mesenteric perfusion. To minimize graft-related infections, the Dacron graft was wrapped using the aneurysm sac, a bovine pericardium patch, and the omentum in cases of infected aneurysms. The mean aortic ACC time ranged from 15 to 30 min. Elective cases required an average of 1.3 units of erythrocyte suspension (ES), while rupture cases required approximately 3 units, supplemented with concentrated human fibrinogen to manage coagulopathy and bleeding.

### 2.8. Treatment Selection

The patient’s clinical status, risk factors, and entire aorta—including its branches and the aneurysm’s anatomical characteristics—were assessed to determine the optimal treatment strategy. Patients were informed about both approaches, with the treatment selection prioritizing informed consent. EVAR eligibility was based on the following anatomical criteria: a proximal aortic neck diameter of 17–32 mm, a non-aneurysmal neck length of ≥15 mm, a proximal neck angulation of ≤60°, minimal thrombus burden within the neck, a distal iliac artery diameter of 8–20 mm, and minimal calcification or tortuosity of the iliac arteries. EVAR was preferred for patients with a high anesthesia risk, multiple comorbidities, prior abdominal surgery, or severe obesity. Conversely, OSR was indicated for patients who did not meet the EVAR criteria and for younger patients with a longer life expectancy [14]. The same criteria applied in rupture cases; however, OSR remained the first-line treatment for hemodynamically unstable patients, particularly when logistical factors, including material availability and procedural feasibility, influenced the decision making.

## 3. Statistical Analysis

All the statistical analyses were performed using SPSS version 28 (IBM Corp., Armonk, NY, USA) and the Jamovi software (version 2.6.19). Categorical variables were presented as frequencies and percentages and analyzed using the chi-square test or Fisher’s exact test, as appropriate. Continuous variables were expressed as the mean ± standard deviation (SD), depending on the normality of the data, which was assessed using the Shapiro–Wilk test. The homogeneity of variances was tested using Levene’s test to determine the suitability of parametric tests. Comparisons between two independent groups were conducted using the Mann–Whitney U test for non-normally distributed variables and the independent samples t-test for normally distributed variables with homogeneous variances. Repeated measurements were analyzed using repeated-measures ANOVA. Additionally, the Yuen test, a robust statistical method that trims extreme values, was utilized to assess group differences in the presence of outliers. A logistic regression analysis was conducted to identify predictors of mortality. Both univariate and multivariate models were constructed to assess the effects of individual and combined variables. The area under the receiver operating characteristic (ROC) curve (AUC) was calculated to evaluate the diagnostic accuracy of the predictive models and to determine the optimal cutoff values. A *p*-value of <0.05 was considered statistically significant for all the analyses.

## 4. Results

A total of 210 patients with AAAs (27 women, 183 men; *p* < 0.01) were included in the study. The patients were categorized into two groups: EVAR (*n* = 163, 77.6%) and OSR (*n* = 47, 22.4%). Their demographic and clinical characteristics, including data on ruptured aneurysms, are summarized in Table 1.

The gender distribution did not differ significantly between the two groups (*p* = 0.983), and sex had no impact on the treatment selection (OR: 0.98, 95% CI: 0.375–2.614; *p* = 0.985). The patients in the EVAR group were significantly older than those in the OSR group (mean difference: 4.80 years, 95% CI: 1.95–7.65; *p* = 0.001). Additionally, the women were significantly older than the men, with a mean age difference of 3.61 years (95% CI: 0.369–6.85; t = 2.31; *p* = 0.031, Yuen’s test).

**Figure 1:**
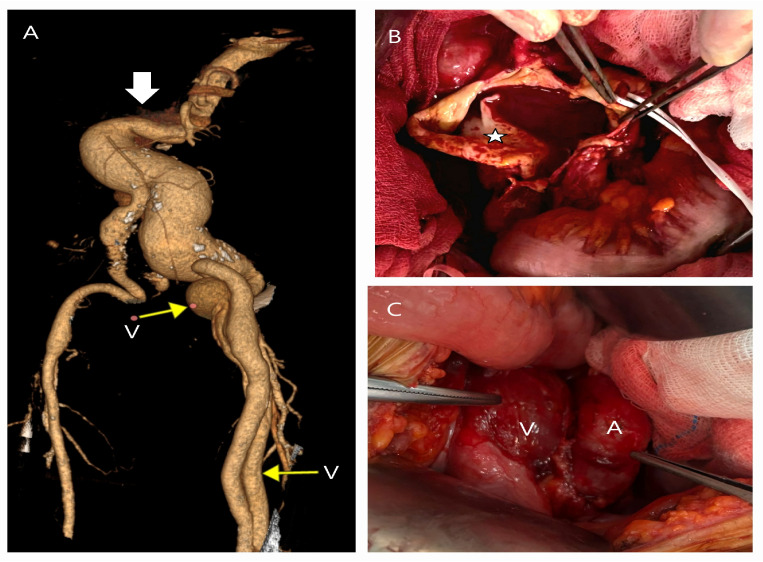
CT and surgical images of an abdominal aortic aneurysm in a 78-year-old male presenting with a pulsating abdominal mass. The patient, a non-smoker with no comorbidities, reported, ’I feel my heart beating in my abdomen’. A 3D CT image (**A**) shows a neck angulation >60° (white arrow) and venous dilatations (V) from the left popliteal to iliac veins (yellow arrows) due to an arteriovenous fistula. Intraoperative findings (**B**) revealed a thin-walled aneurysm with chronic partial dissection (white star) but no thrombus. (**C**) demonstrates severe venous dilation and adhesion between the left common iliac artery (A) and vein (V), complicating surgical exploration.

**Figure 2:**
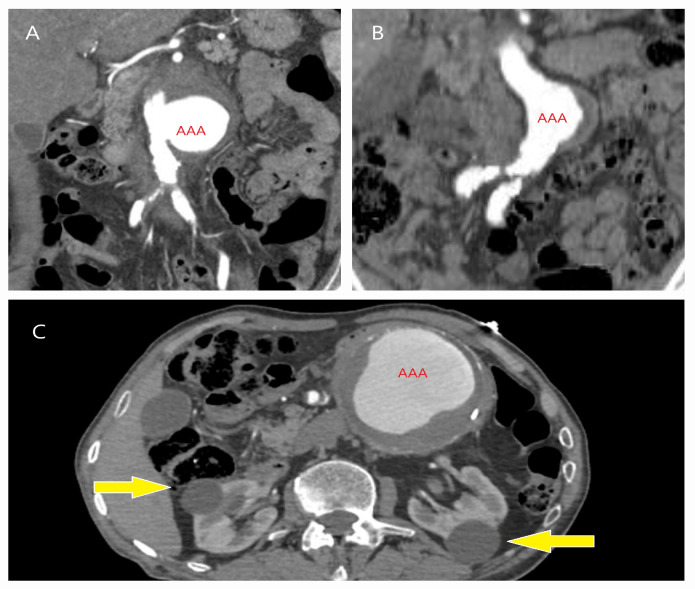
CT images of abdominal aortic aneurysm (AAA) cases incidentally detected during evaluations for unrelated clinical indications. (**A**) A saccular aneurysm was identified in a 60-year-old male during an emergency CT scan after a traffic accident. (**B**) An asymptomatic aneurysm was detected in a 68-year-old hypertensive male with a heavy smoking history (≥2 packs/day) during a prostate cancer evaluation. (**C**) An aneurysm was discovered in a 58-year-old female during a CT scan assessing the vena cava and iliac veins after developing deep vein thrombosis in the left lower extremity while being monitored for cystic kidney disease (yellow arrows) and atrophic kidney.

The most common symptoms included abdominal pain, a pulsating sensation in the abdomen, back pain, and constipation (Figure 1A–C). In several cases, the AAA was incidentally diagnosed during evaluations for unrelated conditions, including cancer treatment (*n* = 10), coronary angiography (*n* = 13), peripheral artery disease (*n* = 17), COVID-19 pneumonia (*n* = 14), trauma assessments (*n* = 4), polycystic kidney disease (*n* = 5), and lumbar MRI (*n* = 2) (Figure 2A–C). Additionally, a 30-year-old patient was diagnosed during pregnancy via ultrasound screening. Among all the patients, only 22 (10.5%) were non-smokers. COPD was present in 153 patients (*n* = 124 EVAR, *n* = 29 OSR), while hypertension was diagnosed in 90.5% (*n* = 152 EVAR, *n* = 38 OSR).

Women had a lower median BMI (25 vs. 26.3 kg/m^2^, *p* = 0.002) and BSA (1.64 vs. 1.95 m^2^, *p* < 0.001) compared to men, resulting in higher ASI values (3.7 vs. 3.4 cm/m^2^, *p* = 0.018). After a BMI adjustment, the mean transverse diameter was 63.9 ± 0.7 mm in women vs. 68.5 ± 0.9 mm in men (*p* < 0.01), while the anteroposterior diameter measured 63.1 ± 0.6 mm in women vs. 68.5 ± 0.8 mm in men (*p* < 0.01). Additionally, women had smaller aortic diameters at the renal level (18.7 ± 6.0 mm) and shorter aneurysm necks (17.9 ± 7.7 mm). These parameters are critical for performing an accurate aortic assessment and selecting the optimal AAA treatment.

Anatomical challenges played a crucial role in the treatment selection. OSR was generally performed in patients who did not meet the EVAR criteria. However, physician-modified fenestrated EVAR was performed in 14 elective patients with short aneurysm necks (10–14 mm) [15]. In the OSR group, suprarenal ACC was required in six patients due to a short aneurysm neck (<15 mm). Table 2 summarizes the number of patients with anatomical challenges, most of whom presented with multiple anatomical complexities.

**Figure 3:**
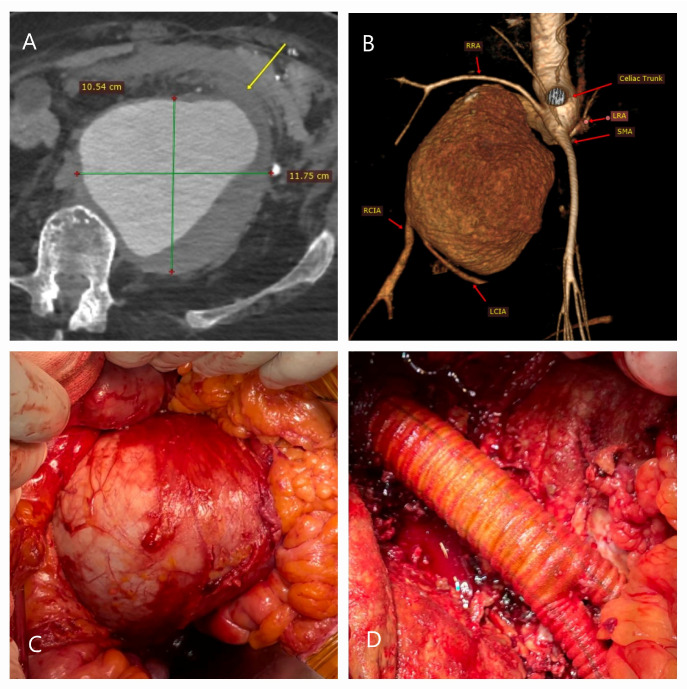
CT and surgical images of a patient with anatomical contraindications for EVAR. A 67-year-old bedridden male with bilateral lower limb amputation and hypertension presented with severe abdominal pain. (**A**) CT angiography revealed a ruptured aneurysm (11.7 × 10.5 cm) with a probable contained rupture (yellow arrow). (**B**) 3D CT reconstruction demonstrated hostile neck anatomy and iliac artery configurations. (**C**) The aneurysm was massive, and (**D**) after excision, a graft was placed, extending to the femoral arteries due to the inability to perform iliac anastomoses. In such cases, rapid surgical intervention is critical, and adjuncts like autotransfusion, aortic occlusion balloons, or Foley catheters may be necessary to ensure hemostasis and improve intraoperative management.

Among the 34 patients with ruptured AAAs (5 females, 29 males), 19 underwent EVAR, while 15 were treated with OSR. OSR was preferred in patients with progressive shock, persistent pain, or anatomical constraints preventing EVAR, as well as in cases where the necessary materials were not immediately available, as they were not stored at our hospital (Figure 3A–D). Conversely, EVAR was favored in patients with a contained rupture and hemodynamic stability (no hemoglobin decrease, adequate blood pressure) if their anatomy was suitable. Although the mean diameter of ruptured aneurysms exceeded 80 mm, the diameter did not influence the treatment selection. Instead, the transverse diameter was a stronger predictor of a rupture than the anteroposterior diameter (OR = 1.05, 95% CI: 1.05–1.2; *p* = 0.030).

In the postoperative period, the OSR group exhibited significantly greater increases from their preoperative levels for their BUN (increase of 16 vs. 5.3 mg/dL; *p* < 0.01) and Cr levels (increase of 0.68 vs. 0.26 mg/dL; *p* = 0.02) compared to the EVAR group. Additionally, the hemoglobin levels declined more markedly in OSR patients, with a reduction of 3 units vs. 1.3 units (*p* = 0.07). Among patients with ruptured AAAs, those treated with OSR demonstrated even higher postoperative laboratory values than the EVAR group, with mean differences of 25 mg/dL for BUN, 1.4 mg/dL for Cr, 78.3 U/L for AST, and 50.4 U/L for ALT.

The overall mortality rate was 9.5% (20/210 patients), with 12 deaths (7.3%) in the EVAR group and 8 (17%) in the OSR group (*p* = 0.085, Fisher’s exact test). The mean time to death was 10.1 days postoperatively (range: 1–28 days). The leading cause of mortality was dialysis-dependent acute renal failure, observed in three EVAR and four OSR patients. Other causes included a myocardial infarction (two EVAR, one OSR), gastrointestinal bleeding (one EVAR, one OSR), an ischemic stroke (two EVAR, one OSR), and mesenteric ischemia (one EVAR patient requiring emergency conversion to open surgery). Additionally, three EVAR patients who required emergency conversion to OSR developed abdominal tamponade, while one OSR patient died due to ileus and sepsis.

Table 3 presents a regression analysis of the mortality predictors. Univariable regression identified the female sex, a rupture, an overweight status, preoperative kidney disease, and postoperative dialysis as significant mortality risk factors. The independent 2.5-fold risk associated with OSR increased to 3.2-fold in the multivariable analysis. Although the model’s significance was moderate due to the small sample size (Nagelkerke’s R^2^ = 0.654), its predictive performance remained meaningful, with an AUC of 0.965 and an accuracy of 0.924 based on a 0.3 cutoff value.

In our study, the mortality was the primary outcome, and Fisher’s exact test yielded a *p*-value of 0.085, suggesting a trend, but not reaching statistical significance (α = 0.05). A post hoc power analysis showed an achieved power of 0.438 (43.8%), below the 0.80 (80%) threshold for adequate statistical power. To reach 80% power, a substantially larger sample size would be required. This limitation underscores the need for larger cohorts in future studies to better assess the impact of the treatment modality on mortality.

The hospital stay was significantly shorter in the EVAR group (mean difference: 6.75 days; ξ = 0.662; *p* < 0.001). Postoperative early complications (<30 days) prolonged hospitalization in both groups. The mean follow-up duration was 27.6 ± 20.2 months (range: 2–80 months) in the EVAR group and 26.3 ± 12.5 months (range: 2–68 months) in the OSR group. Differences in the treatment outcomes became more evident during the postoperative follow-up, particularly regarding the need for reinterventions.

Table 4 summarizes both early and late complications as well as the corresponding treatment procedures.

**Figure 4:**
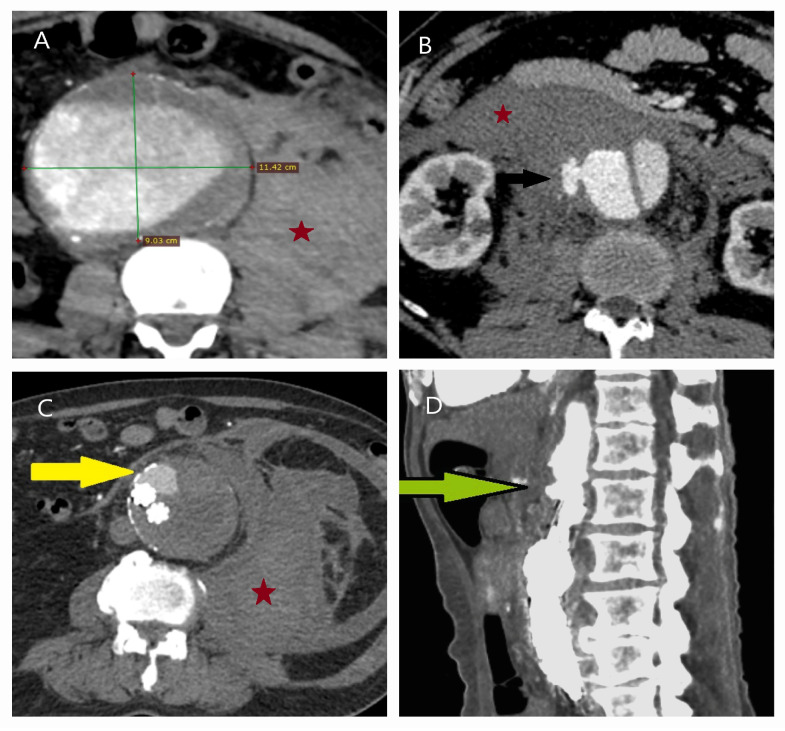
CT images of patients requiring open surgery immediately after EVAR. Three patients—(**A**) a 72-year-old female, (**B**) a 38-year-old male with a dissecting ruptured aneurysm (black arrow indicating the rupture site), and (**C**) a 66-year-old male (yellow arrow indicating a Type 1b endoleak)—developed persistent abdominal distension after EVAR, accompanied by metabolic acidosis. This led to a diagnosis of abdominal tamponade, necessitating open surgery (red stars indicate hematoma). (**D**) A CT image of a 68-year-old male patient who underwent emergency EVAR for rupture but later required open surgery due to superior mesenteric artery (SMA) thrombosis (green arrow) in the postoperative period. A detailed preoperative evaluation of aortic branches is essential before EVAR, and if necessary, mesenteric anastomoses should be performed via open surgery.

**Figure 5:**
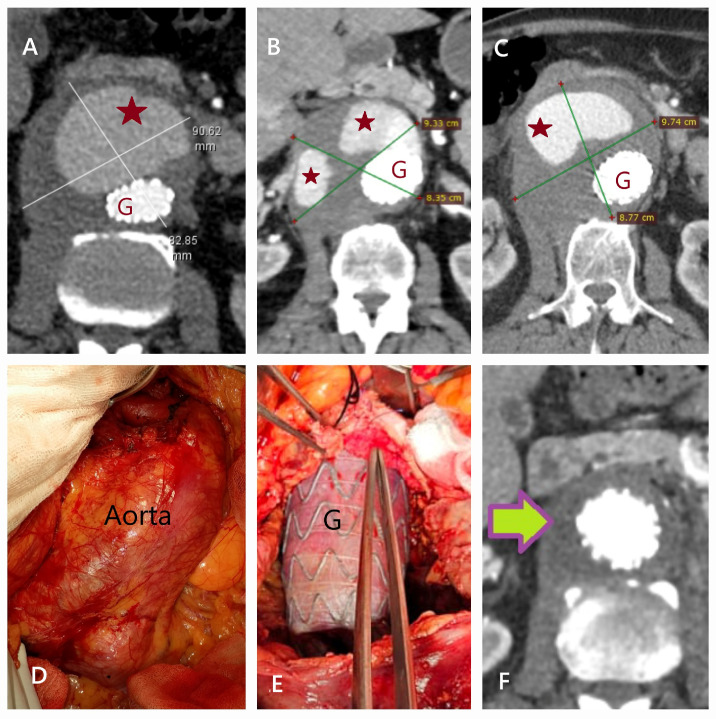
CT and surgical images of Type 1 endoleak. (**A**–**C**) CT scans taken at the first, fourth, and sixth months following EVAR in a 70-year-old male patient demonstrate progressive aneurysm enlargement due to a Type 1 endoleak (red stars indicate contrast agent outside the stent graft; G, stent graft). Despite an intervention with a fenestrated tube graft in the fourth month, aneurysm expansion continued. (**D**,**E**) During open surgery, the aneurysm sac was opened without cross-clamping using an endovascular occlusion balloon. The anterior leakage site (forceps tip) was repaired with pledgeted sutures, and the aneurysm sac was wrapped around the graft. (**F**) The patient’s postoperative second-month CT scan (green arrow) is shown. Stent-preserving surgery is a less invasive alternative to stent explantation in managing EVAR-related complications.

**Figure 6:**
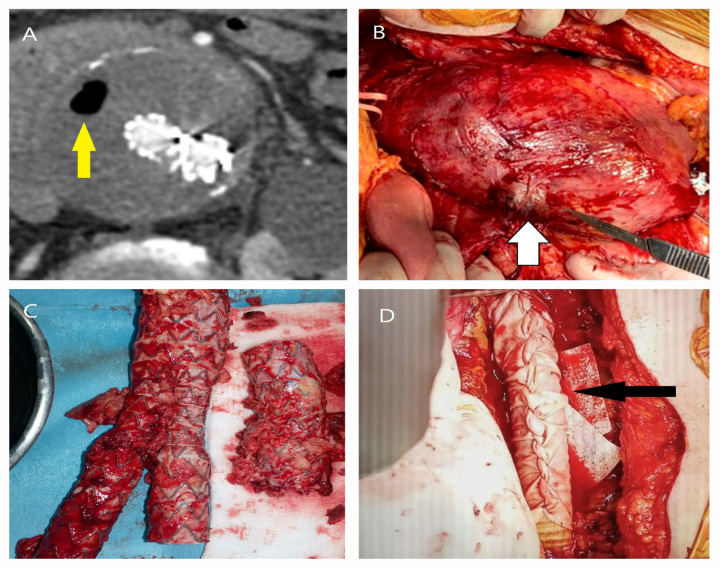
CT and intraoperative images of stent graft infection following EVAR. A 63-year-old male, who underwent EVAR four years ago, was admitted with severe back pain and fever. Blood cultures confirmed Enterococcus faecalis infection. (**A**) CT showed air (yellow arrow) within the aneurysm sac, indicating infection. (**B**) Surgery revealed an inflamed aneurysm sac and an infected protrusion (white arrow). (**C**) The infected stent graft (with iliac limb separation) was removed after proximal occlusion with a balloon. (**D**) A silver-coated, antibiotic-impregnated Dacron graft was placed and wrapped with a pericardial patch (black arrow) and omentum.

Throughout the follow-up period, complications were observed in 26% of EVAR patients and 36% of OSR patients (*p* = 0.191). In the EVAR group, five patients required conversion to open surgery (COS) within the immediate postoperative period (0–1 days), with four cases due to abdominal tamponade and one due to mesenteric ischemia (Figure 4A–D). In the late postoperative period, six additional EVAR patients required OSR due to complications, including a type I endoleak (one in the first year and another in the third year), a type V endoleak (one in the first year), a stent graft infection (one in the first year and another in the fourth year), and graft migration (one in the second year) (Figure 5A–F and Figure 6A–D). In the OSR group, three patients required revision due to bleeding, while one patient who had previously undergone infected EVAR graft removal later required revision for ileus. Other early OSR complications were predominantly surgical site infections, with four patients undergoing vacuum-assisted wound closure (VAC) therapy.

**Figure 7:**
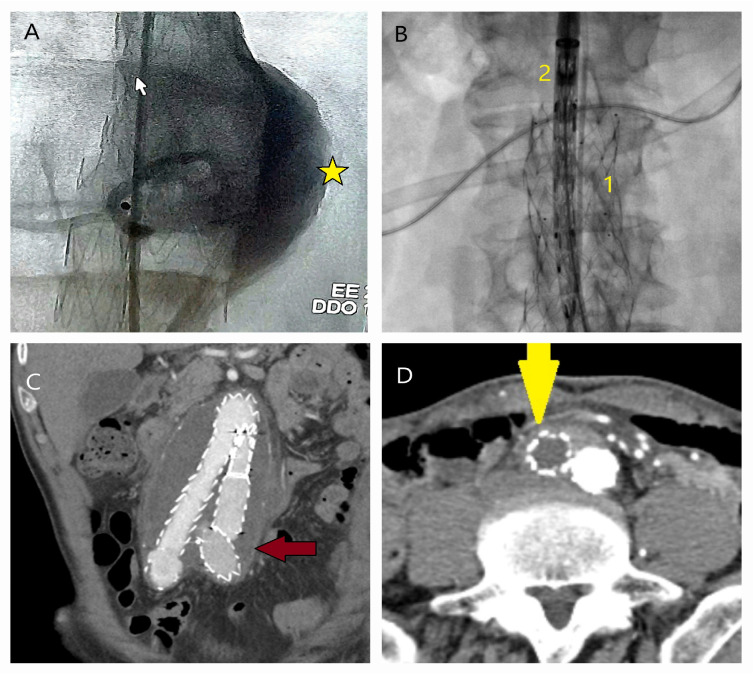
CT and angiographic images demonstrating causes of reintervention following EVAR. (**A**,**B**) In a 75-year-old male patient, a Type 1 endoleak originating from the posterior aspect (yellow star) of the proximal stent graft fixation site (mouse pointer) was treated using the fenestrated technique with proximal extension of a tube graft (1: previous graft; 2: new graft) (**C**) In a 66-year-old male patient, malposition and displacement of the left iliac fixation site (red arrow) were observed. In such cases, aneurysm expansion or infection should be investigated. (**D**) In an 83-year-old female patient, thrombotic occlusion of the iliac stent (yellow arrow) was detected within the first year after EVAR.

Furthermore, 25 EVAR patients required interventional or surgical procedures to manage complications such as endoleaks, graft limb thrombosis, and graft limb integrity failure (Figure 7A–D). These interventions included balloon angioplasty, proximal or distal stent extension, an extra-anatomic bypass, and embolectomy. In the OSR group, four patients underwent surgical repair for a pseudoaneurysm within the first six months, while two patients required an embolectomy for graft thrombosis in the first and third years. These findings may be attributed to the use of modular graft systems in EVAR, the persistence of disease in the affected aortoiliac arteries, and the use of single-piece grafts with suture fixation in OSR. Although the late reintervention rate (>30 days) was higher in the EVAR group, this difference became more evident after the first year, but did not reach statistical significance (HR: 1.2, 95% CI: 0.4–3.6; *p* = 0.754). Within the first year, EVAR-related complications primarily involved graft limb thrombosis and type Ib endoleaks, whereas after two years, type Ia endoleaks became more prevalent. Notably, no cases of type III or type IV endoleaks were observed.

**Figure 8:**
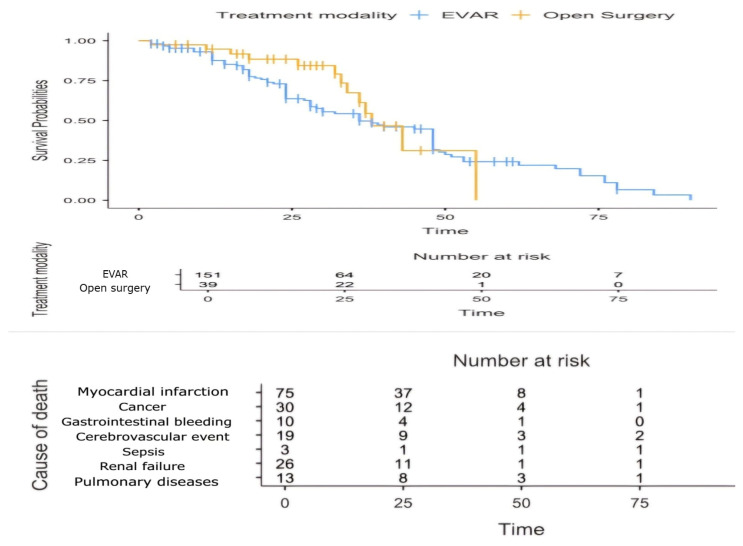
Kaplan–Meier survival curve and causes of death. Kaplan–Meier survival curves comparing EVAR (blue) and Open Surgery (yellow) indicate no significant difference in long-term survival between the treatment modalities. Causes of death are listed by months.

A Kaplan–Meier analysis revealed median survival times of 36 months for the EVAR group and 38 months for the OSR group, with no statistically significant differences between the two treatment modalities (log-rank test, *p* = 0.365), indicating comparable survival outcomes. A Cox regression analysis further supported these findings, demonstrating that neither the treatment modality (*p* = 0.213) nor gender (*p* = 0.951) significantly influenced survival. In contrast, age emerged as a significant predictor, with each additional year associated with a 3.7% increase in the mortality risk (*p* = 0.018). Additionally, non-aneurysm-related causes—including a myocardial infarction, cancer, renal failure, and cerebrovascular events—were the primary contributors to mortality (Figure 8).

## 5. Discussion with a Brief Review of the Literature

This study underscores the essential role of OSR in AAA treatment, particularly in cases where EVAR is unfeasible due to anatomical constraints or material limitations, fails intraoperatively, leads to late stent-related complications, or requires urgent surgical intervention for hemodynamic instability. Although EVAR has become the first-line treatment worldwide, the importance of OSR remains critical and should not be overlooked. To ensure the long-term success of invasive aortic procedures, OSR must be as well understood and mastered as EVAR. Despite the higher proportion of rupture cases in the OSR group, our findings indicate that OSR can be performed with a safety profile comparable to that of EVAR (*p* = 0.085)

**Figure 9:**
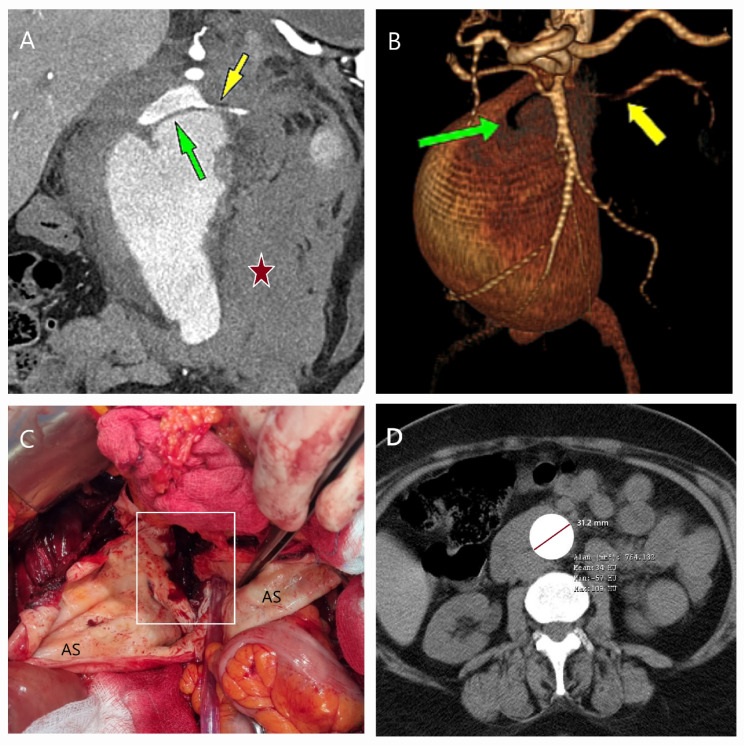
Silent aneurysm growth over six years: CT and surgical images. (**A**,**B**) A 57-year-old female smoker, undergoing hypertension treatment and socially active, presented with severe abdominal pain and syncope. Her poor clinical condition, absence of an aneurysm neck, dissection (green arrow) extending from the left renal artery (yellow arrow), and the presence of a hematoma (red star) necessitated open surgery. (**C**) Intraoperatively, a 7 cm longitudinal rupture (white square) was identified on the posterior aneurysm wall (AS, Aneurysm sac wall). (**D**) Retrospective review of a CT scan from six years earlier, initially performed for urinary stone evaluation, revealed an abdominal aorta measuring only 3 cm. (Alan; which means ‘area’ in English). Unaware of its silent progression, the patient lived for six years without intervention. This case underscores the importance of early detection of aortic pathology across all clinical disciplines and reinforces the need for population-based screening programs.

Although the prevalence of AAAs and AAA-related mortality has declined due to cardiovascular risk management strategies, lifestyle modifications, and opportunistic or national screening programs that increase the rate of elective repair, AAAs remain a global concern [4,16]. Their silent clinical course continues to result in ruptures, causing at least 100,000 deaths annually [17,18]. The non-linear growth rate of aneurysms, coupled with their unpredictable clinical behavior even within the same patient, makes them a persistent burden and a significant risk factor [19] (Figure 9A–D). Since no definitive medical treatment exists to halt the progression of AAAs, EVAR and OSR hold a vital role in their management, effectively serving as rupture prophylaxis [20,21].

Despite nearly a century of unsuccessful attempts to develop a pharmacological treatment for AAAs, management remains limited to risk factor modification, medical surveillance, OSR, and EVAR. Early experimental approaches, such as the cellophane-wrapping technique—credited with extending Einstein’s life—preceded the transformative advent of neoaortic surgery in the 1950s. Four decades later, EVAR, pioneered by Volodos and Parodi, ushered in the “space age” of AAA treatment [22,23,24]. Initially introduced as a low-risk alternative for patients with significant comorbidities, EVAR’s advantages eventually led to its adoption as the primary treatment option. While the number of elective AAA treatments remained stable until 2000, it began to increase following the FDA approval of additional EVAR-related devices [25]. According to SVS Vascular Quality Initiative data, the elective EVAR/OSR ratio over a ten-year period was 5.7, whereas the non-elective ratio was 2.5, with EVAR utilization increasing annually by 0.2% (95% CI: 0.01–0.32) [26]. A global three-year analysis from the International Consortium of Vascular Registries, spanning 11 countries, revealed substantial regional differences in EVAR utilization. In certain regions, EVAR was frequently used for intact AAAs <5.5 cm, reflecting variability in clinical decision making [27]. This disparity may arise from factors such as population density, economic constraints, physician expertise, the hospital capacity, guideline inconsistencies, and disparities in access to medical resources. However, the disproportionately high utilization of EVAR raises concerns not only at the patient level, but also regarding the training of future surgeons.

The widespread adoption of EVAR can be attributed to its minimally invasive nature, feasibility under local anesthesia or sedation, minimized use of blood products, and shorter intensive care unit and hospital stays. However, its most significant advantage has been the reduction in postoperative mortality [28]. The reported mortality rates were 2.9% in the EUROSTAR study (Vallabhaneni et al.; 2721 men, 141 women; mean age: 70.6 years; mean AAA diameter: 56.1 mm) [29], 1.7% in EVAR Trial 1 (Greenhalgh et al.; 983 men, 99 women; mean age: 74 years; mean AAA diameter: 6.5 cm) [30], and 1.2% in the DREAM trial (Prinssen et al.; 159 men, 12 women; mean age: 70.7 years; mean AAA diameter: 60.6 mm) [31]. While early studies suggested OSR as the safer approach for AAA management [32], the findings from these three contemporary trials [29,30,31] indicated that OSR was associated with a mortality rate at least three times higher than EVAR.

Given the cardiopulmonary, renal, and gastrointestinal complications, along with risks associated with bleeding, laparotomy incisions, fluid imbalance, and aortic cross-clamping, OSR is inherently a higher-risk procedure. However, despite these complications, the overall risk rate remains below 10% [33]. Siribumrungwong et al. [34] reported no 30-day mortality, despite performing suprarenal aortic cross-clamping in 22 out of 100 OSR patients. Similarly, in a prospective study by Davidovic et al. [35], the OSR mortality rate was 1.5% (7/450 patients). Twine et al. [36] further emphasized that surgical repair can be performed with a mortality rate below 5% in patients with complex AAAs who are entirely unsuitable for endovascular treatment. Notably, while OSR-related complications are well documented, EVAR patients often share similar risk factors due to their advanced age and high comorbidity burden.

In our study, the overall mortality rate was 9.5% (20/210 patients), with 12 deaths (7.3%) in the EVAR group and 8 (17%) in the OSR group (*p* = 0.085, Fisher’s exact test). Among the EVAR patients, all who died were over the age of 73, including two female patients presenting with a rupture. While no procedure-related mortality was observed, cardio-cerebral, renal, and gastrointestinal complications were the primary contributors. Additionally, three rupture patients succumbed to abdominal tamponade following EVAR. In the OSR group, acute kidney failure requiring dialysis and cardio-cerebral complications were also the leading causes of mortality. However, among the two deceased male patients, one had an EVAR graft infection, while the other experienced stent migration.

A rupture was the strongest predictor of mortality in both groups (Z = 6.23, *p* < 0.001). The combination of hemodynamic instability—including impaired renal function, hypotension, hypoperfusion, and lactic acidosis—along with the effects of an intra-abdominal hematoma and a prolonged operative time significantly increased the mortality risk in both groups. Among rupture patients, four females and three males who underwent EVAR, as well as six males who underwent OSR, did not survive. Although EVAR has become the preferred treatment for ruptured AAAs due to its lower mortality risk [37,38,39], some randomized trials have reported no significant survival difference between EVAR and OSR [40,41]. While lower mortality rates may be expected in centers with immediate access to endovascular resources, delays in hospital admission and the high-risk nature of rupture cases contribute to persistently elevated mortality rates. Regardless of the approach, the mortality remains at 20% at least, though a multidisciplinary team approach can improve the OSR outcomes [42,43,44]. Unresolved challenges include achieving adequate proximal sealing in hypovolemic patients, the 10–20% incidence of abdominal compartment syndrome post-EVAR, the contrast agent burden, and temperature regulation in prolonged procedures. These factors continue to impact the outcomes and warrant further investigation [45,46].

Although meta-analyses highlight the early benefits of EVAR, guidelines lack definitive recommendations regarding the choice between EVAR and OSR [47,48]. In a patient-centered decision-making system, OSR is either considered a secondary option or recommended at an equivalent level to EVAR in individuals with an acceptable risk profile [49,50]. This divergence in perspectives persists in clinical practice, where treatment approaches continue to vary based on individual experience, institutional resources, and regional patient demographics.

While EVAR is preferred for its minimally invasive nature, it necessitates lifelong surveillance and has a higher likelihood of secondary interventions. In contrast, OSR is associated with more stable long-term outcomes, despite its greater perioperative risk. Patients aware of the need for reinterventions and cumulative radiation exposure associated with EVAR may be more inclined to choose OSR, whereas those with a higher surgical risk often opt for EVAR as a safer alternative [51]. Not all high-risk patients are suitable candidates for EVAR, as large aneurysms treated with EVAR due to OSR ineligibility still carry a significant perioperative mortality risk [52,53,54]. To optimize patient selection and treatment strategies, it is essential to establish standardized EVAR indications and ensure their global applicability. Defining clear selection criteria will facilitate a balanced assessment of EVAR’s benefits and limitations while preserving a patient-centered approach [55].

### 5.1. Failure of EVAR

**Figure 10:**
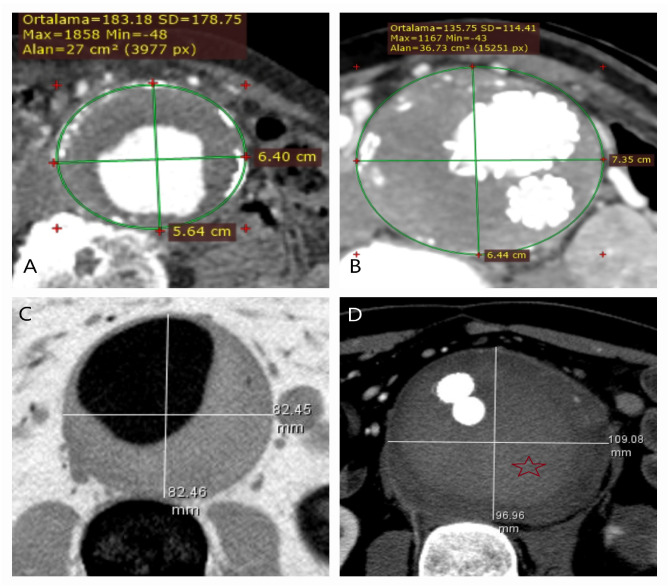
CT images demonstrating aneurysm sac enlargement after EVAR. (**A**,**B**) A 73-year-old male developed limb graft migration due to progressive iliac artery dilation, leading to a Type 1b endoleak and a 1 cm sac expansion two years post-EVAR. (**C**,**D**) A 60-year-old male with coronary artery disease and COPD experienced persistent abdominal pain one year after EVAR, revealing a 2 cm sac enlargement. A hypodense area within the sac, without extraluminal contrast, indicated a Type 5 endoleak (red star). In both cases, continued smoking and poor adherence to antihypertensive therapy contributed to disease progression.

The aging of the aorta, along with the degenerative and genetic pathophysiology of the aortic wall, the persistence of untreated or unmodified risk factors, and the morphological characteristics of the aorta and its branches, significantly influences the success of aortic interventions [56]. EVAR, as a stent graft system, is dependent on adequate sealing zones for fixation within the aorta and appropriate dimensions to ensure proper stent overlap and structural integrity. Consequently, its success in a diseased aorta is determined by the interaction between the physician, the graft system, and patient-specific anatomical factors [57]. Complications often arise due to proximal aortic neck dilation or shortening, iliac artery enlargement, a hostile neck anatomy, persistent flow into the aneurysm sac from lumbar and inferior mesenteric arteries, stent graft erosion, hook fractures, and stent component separation (Figure 10A–D). These factors are frequently interconnected, with one predisposing to another. Given the 20–50% incidence of endoleaks, along with the risks of stent graft migration, graft limb occlusion, stenosis, kinking, and stent graft infection, close postoperative surveillance is essential to prevent sac expansion and a secondary rupture, which may lead to severe complications [58,59].

As long-term follow-up data continue to accumulate, EVAR appears to be gradually losing its superiority over open surgery. According to the 2005 results of EVAR Trial 1 [60], postoperative complications within four years of randomization were observed in 41% of EVAR patients, compared to 9% in the open repair group (OR: 4.9, 95% CI: 3.5–6.8; *p* < 0.0001). Additionally, the cost of EVAR was 1.3 times higher than that of open repair. Seven years after the initial procedure, Mao et al. [61] reported cumulative reintervention risks of 8.3% for EVAR and 3.6% for OSR in Australia, 8.6% and 1.6% in Germany, and 8.3% and 3.1% in the United States, respectively. They also noted that reinterventions occurred earlier in two of these countries. A recently published meta-analysis [62] further demonstrated that the long-term differences between EVAR and OSR become more evident after 8 years. EVAR was associated with significantly higher rates of aneurysm-related mortality (HR: 5.12; 95% CI: 1.59–16.44), secondary interventions (HR: 2.13; 95% CI: 1.69–2.68), an aneurysm rupture (OR: 5.08; 95% CI: 1.11–23.31), and death due to a rupture (OR: 3.57; 95% CI: 1.87–6.80) compared to OSR. These findings highlight concerns regarding the long-term durability of EVAR and the challenges associated with postoperative surveillance.

In our study, 25 EVAR-treated patients required reintervention, primarily due to complications arising after the first year. These were managed using balloon angioplasty, proximal or distal stent extension, an extra-anatomic bypass, and an embolectomy. In patients with type I endoleaks and graft migration, progressive aortic neck dilation, iliac artery expansion, and increased tortuosity were observed. The majority of these patients were persistent smokers with poorly controlled cardiovascular risk factors. A fundamental limitation of EVAR is that residual aortic tissue left untreated does not undergo healing. While the stent system is anatomically compatible, it comprises separate components rather than a fixed unit. Consequently, as the aneurysm enlarges or tortuous iliac arteries impair proper anchoring, EVAR-related complications are likely to persist [63].

### 5.2. Conversion to Open Surgery

Beyond complications that can be managed with minimally invasive interventions, a critical concern with EVAR is the risk or necessity of conversion to open surgery (COS). COS has distinct clinical characteristics, with its prevalence varying by timing (0.8–5.6% in the early period; 0.4–22% in the late period) and its mortality rates ranging from 0% to 28.5%, depending on both the timing and the underlying cause of the conversion [64,65]. In a retrospective study by Becker et al. [66], with a median follow-up of 55 months, COS was found to be multifactorial in origin among 31 patients, the majority of whom had undergone prior reinterventions. Similarly, Mohapatra et al. [67], in an analysis of 102 patients with COS, reported that ruptures and graft infections were associated with a 40% mortality rate.

The variability in COS rates reflects differences in patient compliance with follow-up, while the impact of patients lost to follow-up on the outcomes remains uncertain. The “patient–graft–physician” triad, discussed in the “Failure of EVAR” section, remains a key framework underlying COS [68]. In a cohort study of 4392 patients, Peppelenbosch et al. [69] reported that large aneurysms (≥6.5 cm) were more likely to require COS following EVAR, primarily due to endoleaks (types I, II, and III), device migration, limb occlusion, and aneurysm expansion. With the widespread adoption of EVAR, the instructions for use (IFU) criteria have evolved. However, the off-label use of EVAR, with four out of ten aneurysms expanding within five years post-procedure and a rising incidence of complications, underscores the need for stricter patient selection [70,71]. EVAR performed in complex aortic anatomies—including large neck diameters, short necks, severe neck angulation, and aneurysmal iliac arteries—is associated with a progressive increase in the COS incidence due to the development of aneurysm-related complications over time [72]. Following EVAR, physiological processes within the residual aneurysm persist, leading to aortic remodeling. Consequently, graft displacement and aneurysm expansion may occur. Additionally, persistent collateral flow and graft erosion over time, despite initial attempts at minimally invasive management, remain major contributors to COS [73].

In addition to anatomical factors, Ultee et al. [74] identified an association between COS and both the female sex and a younger age. The inherently higher-risk profile in women, including the increased incidence of intraoperative aortic neck or iliac artery ruptures, as well as leg and colonic ischemia, explains the elevated rate of postoperative complications [75]. However, the treatment strategy for younger patients remains a topic of debate. While meta-analyses have demonstrated the feasibility of EVAR in patients aged 45–60 based on long-term outcomes [76], no graft system can ensure lifelong durability. Therefore, its use in younger patients requires careful consideration.

Among the causes of COS, stent graft infections (SGIs) are the most lethal, carrying the highest mortality risk [77]. The development of SGIs is influenced by the foreign body response, the presence of endoleaks, sac expansion, coil embolization, and repeated interventions [78]. During bacteremia or procedures performed under insufficient sterility, biofilm formation on the graft by microorganisms becomes inevitable [79]. Managing SGIs is particularly challenging and associated with high mortality. As EVAR utilization continues to rise, its incidence is also expected to increase [80]. Although EVAR has demonstrated effectiveness in certain cases of infected aneurysms, its long-term outcomes remain uncertain, necessitating further investigation [81].

In our study, aneurysm-related open surgical conversion occurred in six patients between the first and fourth years following EVAR. All of these patients had initially undergone treatment at local (secondary care) hospitals. One patient with a type Ia endoleak had a pre-existing thrombotic and short-neck aneurysm before undergoing EVAR. Among the two patients with progressive aortic expansion, one had previously undergone fenestrated EVAR, but the endoleak persisted, while the other experienced graft migration due to stent detachment at the fixation site. A stent graft infection developed in one patient during the first year, who ultimately did not survive. In contrast, another patient who developed an SGI in the fourth year was successfully managed with intensive medical therapy.

Despite the increasing recognition of late complications associated with EVAR and the rising frequency of conversion to open surgery, it continues to be the preferred treatment modality [82]. While patient preference remains a key determinant [4], the treatment strategy for patients with progressive aortic expansion ultimately relies on the physician’s clinical judgment and foresight [68].

In addition to a risk assessment, evaluating anatomical suitability is essential—not only for short-term success, but also for optimizing long-term outcomes [83]. Furthermore, strengthening open surgical training and establishing universally standardized EVAR indications through vascular societies and guideline development organizations will be instrumental in improving overall AAA management

## 6. Limitation

This study is limited by its retrospective and single-center design, which may have introduced selection bias when directly comparing the EVAR and OSR outcomes. Our institution performs over 100 aortic procedures annually, and the treatment strategies are strictly based on clinical indications. However, the reduced number of cases included after 2018 due to the impact of the COVID-19 pandemic, along with the heterogeneity in both the number of patients and rupture cases between the EVAR and OSR groups, may have influenced the results. The exclusion of extreme values in the statistical analyses could have further contributed to bias. Additionally, the lack of follow-up data for patients who could not be contacted limits the completeness of our findings. Another constraint is the use of a single stent graft type, preventing direct comparisons with other graft systems and potentially limiting the generalizability of complications and their causality. Moreover, the availability and utilization of EVAR devices vary significantly across different countries due to economic factors and healthcare policies, with some regions lacking access to certain graft systems. This variability contributes to heterogeneous outcomes in the literature, making it difficult to establish universally applicable conclusions. Furthermore, the long-term outcomes of EVAR in younger patients or those with a high life expectancy remain unclear. Further studies are needed to determine EVAR’s durability, late complications, and the optimal follow-up strategies for these patients. Another critical question is why aneurysm growth does not occur in all EVAR-treated patients. Investigating the underlying mechanisms could help refine treatment strategies or lead to the development of novel therapies aimed at optimizing aortic wall protection. Additionally, with the increasing use of EVAR and the need for frequent postprocedural imaging, the potential rise in radiation-induced malignancies remains an open question. Future studies should explore whether this will impose limitations on EVAR usage or necessitate adjustments in surveillance protocols. Given these uncertainties, prospective randomized trials are essential to standardize patient selection criteria, evaluate the impact of different stent graft designs, and determine the most effective pharmacological and interventional strategies for long-term aortic wall preservation.

## 7. Conclusions

OSR and EVAR are complementary treatment strategies that should be selected based on patient-specific factors. While EVAR is favored for its minimally invasive nature and lower perioperative risk, its higher rates of reintervention, graft-related complications, and aneurysm-related mortality make its long-term durability a matter of debate. Despite its greater initial surgical burden, OSR remains a reliable and often necessary intervention, particularly in managing EVAR-related complications. As the OSR case volumes decline, the reliance on EVAR is expected to increase. However, in centers where OSR is not performed, the management of EVAR complications may become increasingly challenging, raising concerns about patient outcomes and surgical expertise. Further research is needed to refine patient selection criteria, investigate the mechanisms behind aneurysm sac enlargement in some EVAR-treated patients, and evaluate the long-term implications of frequent imaging surveillance, including the potential rise in radiation-induced malignancies. Maintaining expertise in both OSR and EVAR is crucial to ensuring optimal patient outcomes, rather than allowing one approach to replace the other. A balanced, multidisciplinary strategy will be key in the future of aortic aneurysm management.

## Figures and Tables

**Table 1:** The demographic and preoperative clinical characteristics of all patients, as well as those with rupture.

Patient Demographics		Treatment Modality			Patients with Rupture		
		EVAR *(n* =163)	OSR (n = 47)	*p*	EVAR *(n* = 19)	OSR *(n* = 15)	*p*
Female/Male		21/142	6/41	0.983	5/14	0/15	0.042
Age (years)		73.5 ± 8.7	68.7 ± 8.7	0.001	72.3 ± 12.9	71.4 ± 6.0	0.790
Smoker		148 (90.8%)	40 (85.1%)	0.263	17	13	0.603
Diabetes mellitus		45 (27.6%)	11 (23.4%)	0.566	5	5	0.978
Hypertension		152 (93.3%)	38 (80.9%)	0.011	18	13	0.409
COPD		124 (76.1%)	29 (61.7%)	0.051	13	11	0.755
* Cardiac disease		113 (69.3%)	28 (59.6%)	0.210	16	9	0.112
** Renal disease		53 (32.5%)	19 (40.4%)	0.327	12	12	0.285
*** Extracoronary arteriopathy		28 (17.2%)	9 (19.1%)	0.755	1	4	0.080
Cancer		20 (12.3%)	5 (10.6%)	0.761	3	1	0.397
Saccular aneurysm		9 (5.5%) (Tube graft no iliac limbs)	1 (2.2%)	0.103	-	-	-
Mycotic aneurysm		0	2 (4%)	NA	-	-	-
Incidental diagnosis		57 (35%)	9 (19.1%)	<0.05	-	-	-
Diameter (mm)	Anteroposterior	66.9 ± 12.9	68.8 ± 17.5	0.567	81.0 ± 14.4	81.0 ± 21.7	1
	Transvers	67.5 ± 12.9	71.5 ± 16.1	0.042	82.7 ± 12.0	83.8 ± 19.5	0.478
	Level of the renal artery	21.9 ± 3.6	22.8 ± 6.3	0.148	22.5 ± 4.5	23.8 ± 5.2	0.455
Glucose, mg/dL		124 ± 40	129 ±42	0.483	144 ± 56	147 ± 53	0.880
Hemoglobin, g/dL		13.4 ± 2.8	13.3 ± 2.3	0.697	10.6 ± 2.4	10.8 ±2.5	0.812
Hematocrit, %		42.5 ± 27.3	40.0 ± 8.3	0.538	32.1 ± 7.0	32.7 ± 7.7	0.818
WBC, 10^3^/µL		9.4 ± 3.4	10.4 ± 4.7	0.423	12.1 ± 4.7	14.8 ± 5.2	0.123
ALT, U/L		22.2 ± 17.9	29.7 ± 24.9	0.078	24.8 ± 18.7	46.2 ± 36.8	0.089
AST, U/L		27.0 ± 20.0	40.2 ± 35.4	0.091	37.5 ± 23.9	61.2 ± 55.2	0.286
BUN, mg/dL		23.5 ± 11.7	29.6 ± 15.6	0.054	32.5 ± 17.5	42.3 ± 15.1	0.096
Cr, mg/dL		1.1 ± 0.6	1.3 ± 0.8	0.168	1.7 ± 0.9	2.1 ± 1.0	0.280
LDL, mg/dL		113 ± 32	117 ± 31	0.381	88 ± 34	111 ± 36	0.068
D-dimer, mg/L		5.4 ± 4.7	5.9 ± 6.1	0.213	15.2 ± 4.4	12.5 ± 4.7	0.104
CRP, mg/L		33.6 ± 37.2	47.4 ± 58.5	0.653	100.3 ± 33.5	106.0 ± 55.3	0.918
HbA1c, %		6.0 ± 0.8	6.1 ± 1.1	0.546	6.1 ± 1.0	6.6 ± 1.3	0.238

ALT: alanine aminotransferase; AST, aspartate aminotransferase; BUN, blood urea nitrogen; COPD, chronic obstructive pulmonary disease; Cr, Creatinine; CRP, C-reactive protein; LDL, low-density lipoprotein; WBC, white blood cell. NA: not applicable. * Cardiac disease: Includes all patients with any form of cardiac condition; ** Renal disease: Includes all patients with creatinine levels above 1.2 who are under nephrology follow-up, including those undergoing dialysis; *** Extracoronary arteriopathy: Refers to patients with carotid artery disease or peripheral artery disease.

**Table 2:** The number of patients with anatomical challenges.

Anatomical Challenges	EVAR		OSR	
	Female	Male	Female	Male
Neck diameter of <17 mm or >32 mm	3	3	5	5
Neck length of <15 mm	2	12	3	8
Neck angulation of > 60^0^	1	3	5	10
Neck thrombus > 50%	1	2	0	4
Distal iliac artery diameter of <8 mm or >20 mm	2	5	1	7
Small common femoral artery (<6 mm)	2	1	1	5

**Table 3:** The regression analysis of mortality predictors.

	Univariable			Multivariable		
Predictor	Estimate	*p*	OR (95% CI)	Estimate	*p*	OR (95% CI)
Open surgery	0.9	0.053	2.5 (0.98–6.7)	1.14	0.212	3.2 (0.5–18.8)
Rupture	2.7	<0.001	15.0 (5.3–41.6)	1.7	0.045	5.8 (1.0–32.3)
Female	1.24	0.022	3.4 (1.2–9.9)	2.8	0.018	16.7 (1.7–88.0)
Age	0.01	0.659	1.01 (0.96–1.07)	0.05	0.398	1.04 (0.95–1.15)
Aortic size index (cm/m^2^)	1.06	<0.001	2.9 (1.6–5.0)	1.3	0.019	3.7 (1.2–10.9)
BMI > 28 (kg/cm^2^)	0.15	0.080	1.15 (0.98–1.36)	0.81	0.003	2.2 (1.3–3.9)
Cardiac disease	0.73	0.206	2.1 (0.67–6.5)	2.1	0.064	8.4 (0.8–80.0)
Preoperative creatinin >1.8 mg/dL	1.8	<0.01	6.2 (2.3–16.3)	0.3	0.718	1.5 (0.2–9.1)
Nephropathy with dialysis	3.1	<0.001	23.5 (8.0–69.0))	1.4	0.101	4.2 (0.7–24.0)
Presence of complication	3.0	<0.001	20.5 (5.7–67.0)	3.0	0.003	20 (2.6–127.0)

BMI, body mass index; OR, odds ratio.

**Table 4:** Early and late complications in both groups.

Complications	EVAR	Treatment	OSR	Treatment
Abdominal tamponade	4	Open surgery	3	Re-exploration
Mesenteric ischemia/ileus	1	Open surgery	1	Re-exploration-
Paralytic ileus	-	-	3	Mobilization
Sepsis	1	Sepsis filter and antibiotics	2	Sepsis filter and antibiotics (One patient with stent graft infection)
Femoral bleeding or pseudoaneurysm	3	Revision/aneurysmectomy	6	Revision/aneurysmectomy
Graft limb thrombosis/kinked	5	Embolectomy/balloon Angioplasty/cross-femoral bypass	2	Embolectomy
Graft infection	2	Stent explantation		
Graft migration	1	Stent explantation		
Type Ia endoleak	6	Stent-sparing surgery (2 cases); Proximal stent extension (4 cases)		
Type Ib endoleak	10	Stent extension and balloon angioplasty		
Type II endoleak	3	Embolization		
Type V endoleak	1	Stent-sparing surgery		

EVAR: Endovascular Aneurysm Repair; OSR: Open Surgical Repair.

## Data Availability

The imaging materials used in this review are stored in the authors’ archives, and no data have been obtained from external sources or previously published materials. Imaging data are available upon request from the corresponding author.

## Abbreviations

AAA	Abdominal aortic aneurysm
AUC	Area under the curve
OSR	Open surgical repair
COS	Conversion to open surgery
COPD	Chronic obstructive pulmonary disease
CT	Computed tomography
IFU	Instructions for use
MRI	Magnetic resonance imaging
OR	Odds ratio
ROC	Receiver operating characteristic
